# Supplementary material for: Tuning Optoelectronic Properties and Photoelectrochemical Performance of β‐TaON via Vanadium Doping
Source: Small. 2026 Feb 13;22(21):e10276. doi: 10.1002/smll.202510276 (PMC13081105; doi:10.1002/smll.202510276)
Supplement: Supplementary file 1 — Supporting File: smll72835‐sup‐0001‐SuppMat.docx. [file SMLL-22-e10276-s001.docx]

Supporting Information

Tuning Optoelectronic Properties and Photoelectrochemical Performance of β-TaON via Vanadium Doping

Mirabbos Hojamberdiev^a,b,*^, Ronald Vargas^c,d^, Lorean Madriz^c,d^, Dilshod Nematov^e,f^, Ulugbek Shaislamov^g^, Hajime Wagata^h^, Yuta Kubota^b^, Kunio Yubuta^i^, Katsuya Teshima^i^, and Nobuhiro Matsushita^b^

*^a^Mads Clausen Institute, University of Southern Denmark, Alsion 2, 6400 Sønderborg, Denmark*

*^b^Department of Materials Science and Engineering, School of Materials and Chemical Technology, Institute of Science Tokyo, 2-12-1 Ookayama, Meguro, Tokyo 152-8550, Japan*

*^c^Instituto Tecnológico de Chascomús (INTECH), Consejo Nacional de Investigaciones Científicas y Técnicas (CONICET), Avenida Intendente Marino, Km 8,2, B7130IWA Chascomús, Provincia de Buenos Aires, Argentina*

*^d^Escuela de Bio y Nanotecnologías, Universidad Nacional de San Martín (UNSAM), Avenida Intendente Marino, Km 8,2, B7130IWA Chascomús, Provincia de Buenos Aires, Argentina*

*^e^Physical−Technical Institute, National Academy of Sciences of Tajikistan, Dushanbe
734063, Tajikistan*

*^f^School of Optoelectronic Engineering & CQUPT-BUL Innovation Institute, Chongqing University of Posts and Telecommunications, Chongqing 400065, China*

*^g^Center for Development of Nanotechnology at the National University of Uzbekistan, University Str. 4, 100174 Tashkent, Uzbekistan*

*^h^Department of Applied Chemistry, School of Science and Technology, Meiji University, 1-1-1-D615 Higashimita, Tama, Kawasaki 214-8571, Japan*

*^i^**Institute for Aqua Regeneration, Shinshu University, 4-17-1 Wakasato, Nagano 380-8553, Japan*

*Corresponding author at: Mads Clausen Institute, University of Southern Denmark, Alsion 2, 6400 Sønderborg, Denmark. E-mail address: [mirabbos@mci.sdu.dk](mailto:mirabbos@mci.sdu.dk)

**Density functional theory (DFT) calculations**

First-principles density functional theory (DFT) calculations were performed to investigate the effects of vanadium doping on the structural, electronic, optical, and phonon properties of β-TaON, to assess its potential for photocatalytic applications. The necessity of this computational approach is further supported by analogous results previously reported for SrTiO_3_, where vanadium incorporation was shown to reduce the band gap and suppress recombination centers, thereby significantly enhancing photocatalytic activity.^[1]^ In this study, all calculations were performed using the Vienna Ab initio Simulation Package (VASP).^[2]^ The interactions between the ionic nuclei and valence electrons were described using the PAW method, with convergence thresholds of 10^-6^ eV for total energy and 0.01 eV Å^-1^ for forces. All calculations employed a plane-wave cutoff of 800 eV and a 4×4×4 Monkhorst–Pack k-point grid, which were selected based on convergence tests performed for pristine β-TaON using a standard GGA functional (Figures S6a and S6b). All calculations were spin-polarized to account for the possible magnetic behavior introduced by vanadium doping. To simulate vanadium doping in the β-Ta_1-x_V_x_ON system, appropriate supercells were constructed by partially substituting Ta atoms with V atoms at various concentrations. In contrast to a previous theoretical study that focused solely on Ta_0.25_V_0.25_ON,^[3]^ the present study explores a wider range of vanadium concentrations (Ta_0.95_V_0.05_ON, Ta_0.90_V_0.10_ON, Ta_0.85_V_0.15_ON, Ta_0.80_V_0.20_ON, and Ta_0.75_V_0.25_ON). This enables a more comprehensive investigation of nonlinear correlations between vanadium concentration and the resulting changes in structural, electronic, optical, and thermodynamic properties. Furthermore, the phonon density of states was analyzed to assess the dynamic stability of the doped systems.

Given the high computational cost associated with hybrid functional methods for large supercells, structural optimizations and the evaluation of phonon and thermodynamic properties (in the temperature range of 0 - 1000 K) were performed using the strongly constrained and appropriately normed (SCAN) meta-GGA functional,^[4]^ which has shown excellent performance in describing the structure and ground-state properties of transition metal compounds. Phonon spectra and the corresponding thermodynamic quantities (Helmholtz free energy, entropy, and heat capacity) were computed using the finite-displacement method as implemented in the Phonopy package,^[5]^ based on the SCAN-optimized structures. To accurately describe the electronic structure, optical properties, and effective masses of electrons and holes, the modified Tran–Blaha Becke–Johnson (TB-mBJ) potential^[6]^ was employed. Compared to the HSE06 hybrid functional,^[7–9]^ TB-mBJ offers a computationally efficient alternative that, for this class of oxynitride materials, provides band gap estimates close to those obtained with HSE06, along with reliable dielectric functions and absorption coefficients, particularly for oxide and oxynitride systems with complex dopant configurations and large supercells.

**References:**

[1] H. Bantawal, U.S. Shenoy and D.K. Bhat, Vanadium-doped SrTiO_3_ nanocubes: Insight into role of vanadium in improving the photocatalytic activity, *Appl. Surf. Sci.* 513 (2020) 145858.

[2] G. Kresse and J. Furthmüller, Efficiency of ab-initio total energy calculations for metals and semiconductors using a plane-wave basis set, *Compt. Mater. Sci.* 6 (1996) 15–50.

[3] M. Harb and L. Cavallo, Suitable Fundamental Properties of Ta_0.75_V_0.25_ON Material for Visible-Light-Driven Photocatalysis: A DFT study, *ACS Omega* 1 (2016) 1041–1048.

[4] J. Sun, A. Ruzsinszky and J.P. Perdew, Strongly Constrained and Appropriately Normed Semilocal Density Functional, *Phys. Rev. Lett.* 115 (2015) 036402.

[5] A. Togo and I. Tanaka, First principles phonon calculations in materials science, *Scrip. Mater.*  108 (2015) 1–5.

[6] F. Tran and P. Blaha, Accurate Band Gaps of Semiconductors and Insulators with a Semilocal Exchange-Correlation Potential, *Phys. Rev. Lett.* 102 (2009) 226401.

[7] J. Heyd, G.E. Scuseria and M. Ernzerhof, Hybrid functionals based on a screened Coulomb potential, *J. Chem. Phys.* 118 (2003) 8207–8215.

[8] A.V. Krukau, O.A. Vydrov, A.F. Izmaylov and G.E. Scuseria, Influence of the exchange screening parameter on the performance of screened hybrid functionals, *J. Chem. Phys.* 125 (2006) 224106.

[9] J. Paier, M. Marsman, K. Hummer, G. Kresse, I.C. Gerber and J.G. Ángyán, Screened hybrid density functionals applied to solids, *J. Chem. Phys.* 124 (2006) 154709.


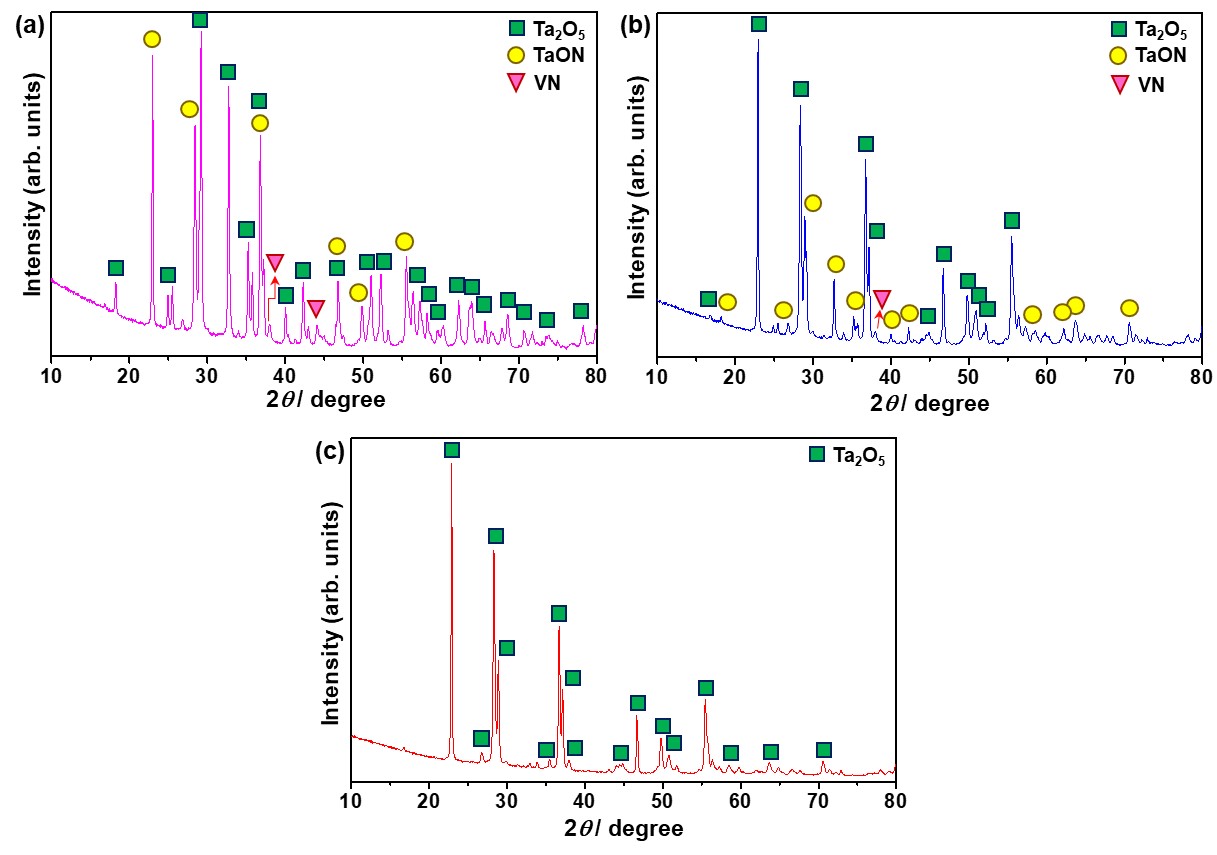


**Figure S1.** X-ray diffraction patterns of the samples synthesized using V_2_O_4_ (a), VN (b), and metallic V (c) as the vanadium precursor (25 at.% V) under identical ammonolysis conditions.


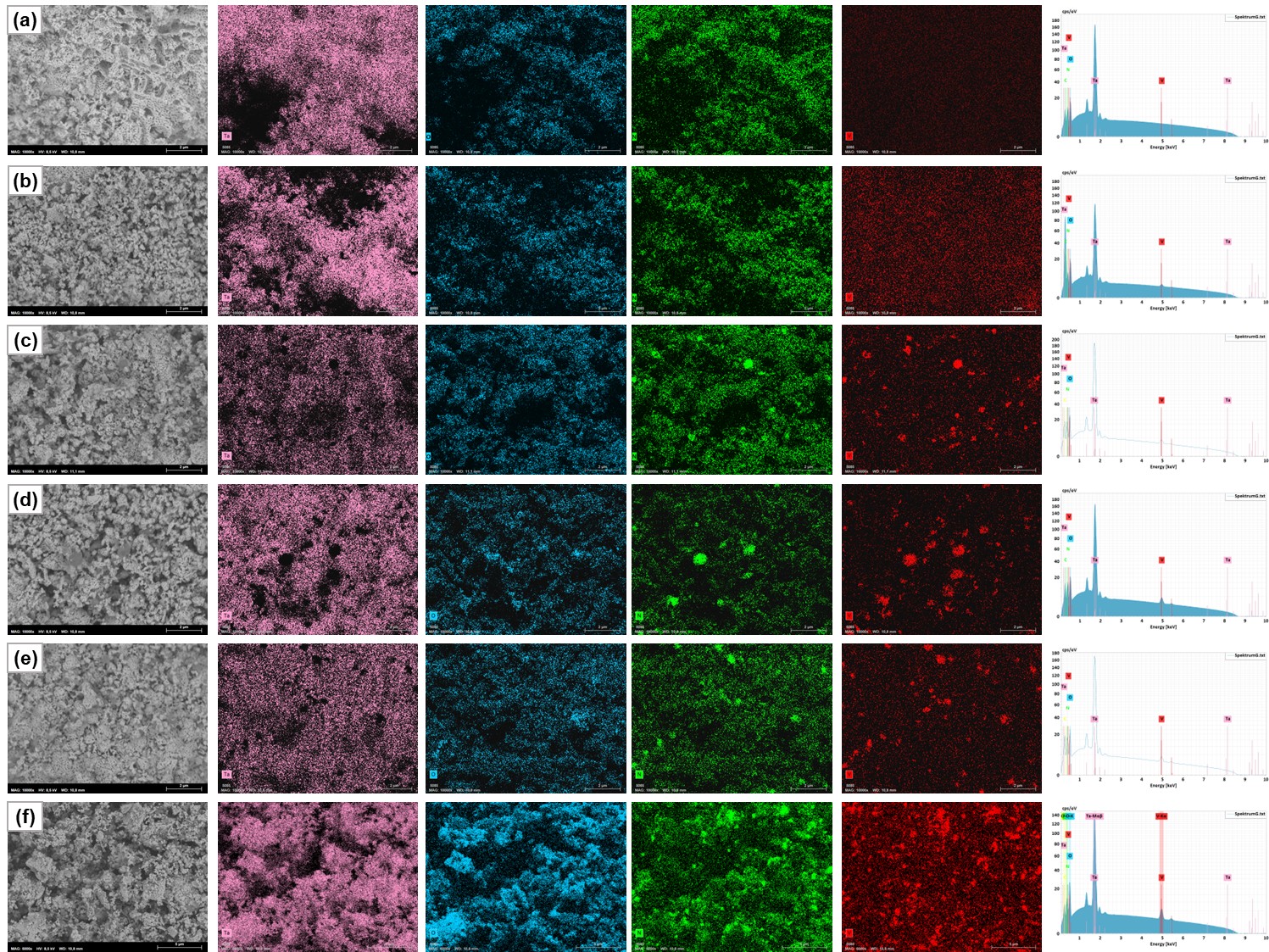


**Figure S2.** EDS element mapping images and spectra of pristine and V-doped β-TaON samples with different vanadium contents: (a) 0 at.%, (b) 5 at.%, (c) 10 at.%, (d) 15 at.%, (e) 20 at.%, and (f) 25 at.%. Grey images, Ta mapping images (pink), O mapping images (light blue), N mapping images (green), V mapping images (red), and EDS spectra.


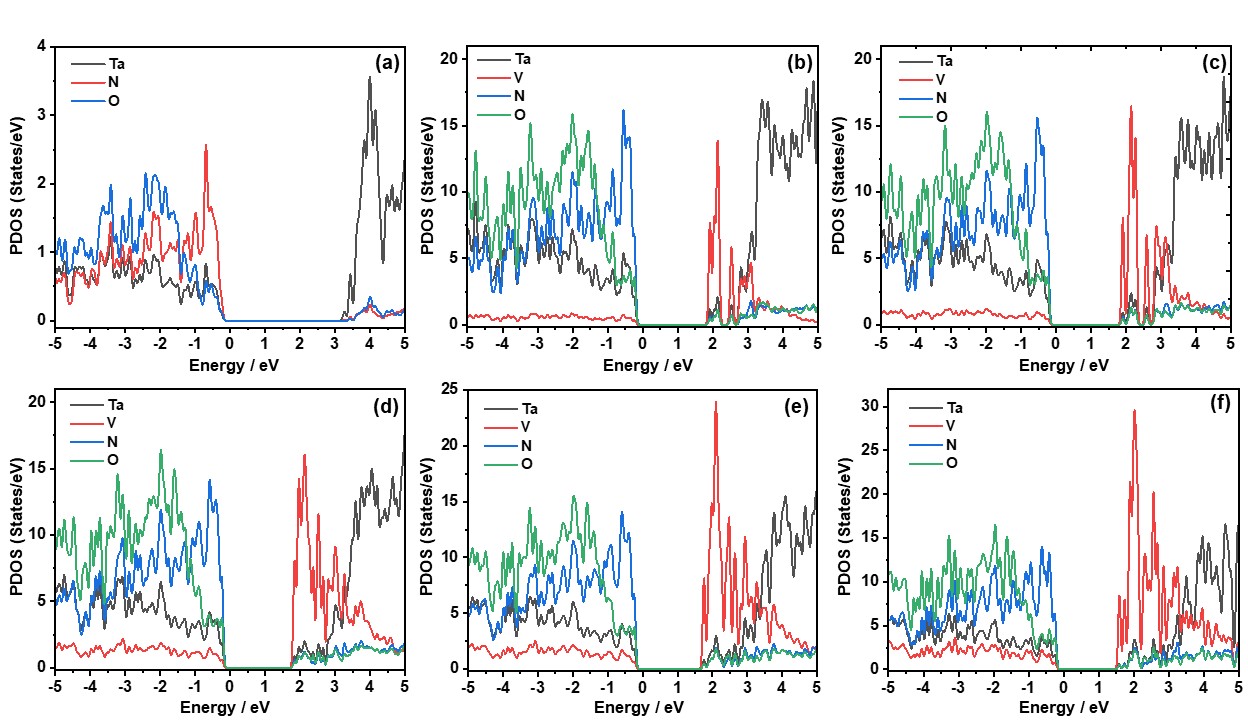


**Figure S3.** Partial density of states (PDOS) diagrams of pristine and V-doped β-TaON samples with different vanadium contents: (a) 0 at.%, (b) 5 at.%, (c) 10 at.%, (d) 15 at.%, (e) 20 at.%, and (f) 25 at.%.


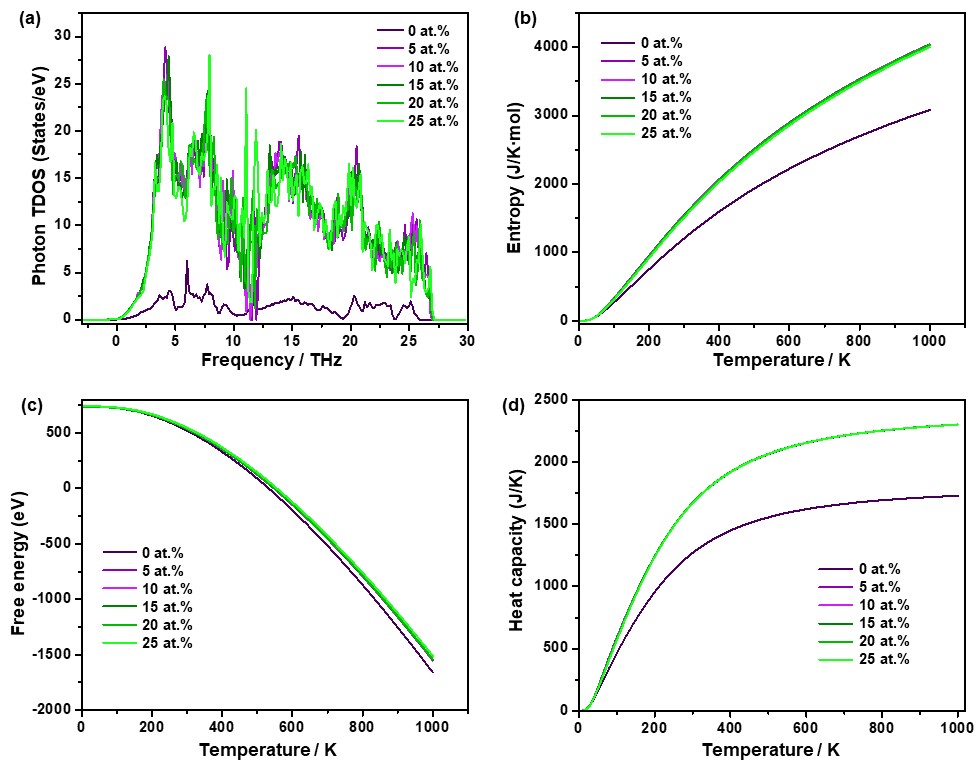


**Figure S4.** Phonon density of states (a), entropy (b), Helmholtz free energy (c), and constant-volume heat capacity (d) of pristine and V-doped β-TaON samples with different vanadium contents: 0 at.%, 5 at.%, 10 at.%, 15 at.%, 20 at.%, and 25 at.%.


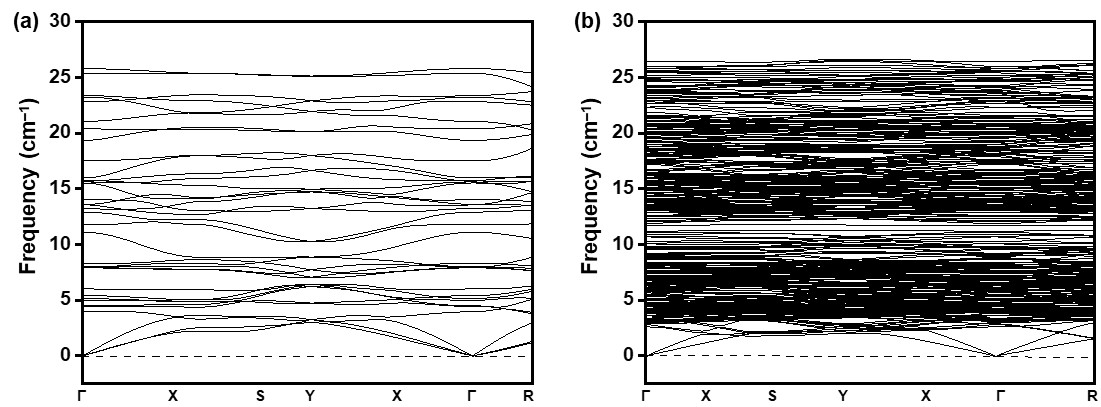


**Figure S5.** Phonon dispersion curves of (a) pristine β-TaON (TVON0) and (b) 10 at.% V-doped β-TaON (TVON10) calculated along high-symmetry paths in the Brillouin zone.

**Figure S6.** Convergence tests for pristine β-TaON: (a) total energy as a function of plane-wave cutoff energy and (b) total energy as a function of k-point density, comparing Γ-centered and Monkhorst–Pack schemes.
